# Children Malnutrition in Northwestern, Central and Southern Regions of Iran: Does Geographic Location Matter?

**DOI:** 10.5539/gjhs.v6n4p36

**Published:** 2014-04-02

**Authors:** Sakineh Nouri Saeidlou, Fariba Babaei, Parvin Ayremlou

**Affiliations:** 1 Food and Beverage Safety Research Center, Urmia University of Medical Science, Urmia, Iran; 2 Urmia University of Medical Science, Urmia, Iran

**Keywords:** underweight, stunting, wasting, under 5 years children, Iran

## Abstract

**Introduction:**

Malnutrition is one of the most important morbidity and mortality causes in children. In comparison with healthy children malnourished children are at higher risk of illness and death as 60 percent of more than 7 million deaths in children aged less than five years are attributed to the malnutrition. The present study is intended to determine the prevalence of malnutrition in West Azerbaijan and compare with Kermanshah and Isfahan provinces.

**Materials and Methods:**

The current survey is a cross-sectional study which is conducted with the aim of determining the nutritional status of children aged less than five years in three West Azerbaijan, Kermanshah and Isfahan provinces using ENA software and has been performed since 16th until 30th October, 2011 with the cooperation of the Office of Community Nutrition Improvement and the United Nations Children’s Fund (UNICEF). Research data are collected by questionnaire and according WHO index, percentage of children with malnutrition (underweight, wasting, stunting) were calculated. Chi-square test was used to assess the relationship between variables and malnutrition.

**Results:**

The rate of underweight, stunting, and wasting in West Azerbaijan was 2.3%, 7.3% and 1.4%, respectively. Wasting rate in boys was higher than in girls while stunting and underweight were more common in girls but differences were not significant. Results showed that the percentage prevalence of stunting in rural areas was higher than in urban areas, and this difference was significant. (p<0.03) prevalence of overweight in West Azarbijan, Kermanshah and Isfahan was 5.1%, 4.5% and 3.7%, respectively. Also, Prevalence of obesity in West Azarbijan, Kermanshah and Isfahan was 1.3%, 0.7% and 0.1%, respectively.

**Conclusion:**

Given the differences between various provinces and regions of the country which are as a result of the differences between the levels of development in these areas, the necessity of designing and implementing targeted strategies are required for different areas.

## 1. Introduction

Malnutrition in children is the main contributor to global burden of disease and causes heavy health expenditures in developing countries especially in Asia and is associated with about half of all child deaths in worldwide (Veghari, 2012; Sharifzadeh et al., 2010). Malnutrition is one of the most important causes of children’s improper physical and mental development of children (Das & Rahman, 2011; Javanparast et al., 2009). Malnutrition also increases susceptibility and incidence of infections and is associated with diminished response to vaccines (Park et al., 2012; Rodriguez-Llanes et al., 2011). One in every five children in the developing world is malnourished, and poor nutrition is associated with half of all child deaths worldwide (Psaki et al., 2012).

Globally, about one of four children who are aged less than five years (26 percent in 2011). According to an estimation 80 percent of the world`s 165 million stunted children are living in just 14 countries. Among all societies, the southern Asian countries obtained the highest success in reducing the prevalence of nutritional stunting in children under 5 years old. However, given the population of this region, a large number of children are still suffering from stunting (Milman, 2005). In May 2012 WHO agreed on a new target: reducing the number of stunted children under the age of five up to 40 percent until 2025. Globally, in 2011, an estimated 101 million children aged less than five years were underweight, or approximately 16 percent of children under five years. Underweight prevalence is highest in South Asia, which has a rate of 33 percent, followed by sub-Saharan Africa, with 21 percent. South Asia has 59 million underweight children, whole sub-Saharan Africa has 30 million. Moderate and severe wasting represents an acute form of under nutrition; children who suffer from it face a significant increased risk of death. Globally, in 2011, 52 million children children aged less than five years were moderately or severely wasted, an 11 percent decrease from the estimated figure of 58 million in 1990 (Park et al., 2012). More than 29 million children aged less than five years, namely, five percent, suffer from severe wasting. malnutrition is one of the major health and nutritional problems in Iran (Sharghi et al., 2011). Results of last civil study in Iran in 1998 show that 5 percent of children suffer from moderate to severe wasting, 15 percent of children under five suffer from moderate to severe stunting and 11 percent of children are under-weight (Sheikholeslam et al., 2004). According to a survey in West Azerbaijan, 8.7%, 7.5% and 4.3% of the children aged less than five years suffered from stunting, wasting, and underweight respectively(Farrokh-Eslamlou 2013). Nutritional status of children under five years is an important indicator that shows the Public health and this survey can lead to create a solution for the preventing and controlling methods of malnutrition. This study aimed to determine the prevalence of malnutrition (underweight, stunting, wasting, and overweight and obesity) in children aged less than 5 years old in West Azerbaijan province and compare it with similar studies in the Esfahan and Kermanshah provinces.

## 2. Methods

The current study is a cross-sectional and analytical study conducted for assessing the nutritional status of children aged less than five years old in three provinces of West Azerbaijan, Kermanshah and Isfahan using ENA and has been performed since 16th until 30th October, 2011 with the cooperation of the Office of Community Nutrition Improvement and the United Nations Children’s Fund (UNICEF). Institutional Review Board approved this study. The statistical population included all 6-59 months children residing in the cities and villages of these areas that among them, 2525 children were selected by cluster sampling. After collecting data, the information of the questionnaire was entered into the statistical ENA software and then, given the WHO indexes, the percentage of children suffering from malnutrition (underweight, stunting, wasting, and overweight and obesity) was calculated. In this study, children with mentally and physically problems were excluded from study and replaced by another children. Cluster sampling was used for selecting the cases and Sample size was calculated with 5% confidence interval through ENA software. 902 children were determined for West Azerbaijan, 794 and 829 cases for Isfahan and Kermanshah, respectively. Data were collected from measuring height and weight and arm circumference of the children, completing the questionnaire and interviews with mothers or caregivers of children. Scales used in this study was a single pan balance with the maximum capacity of 150 kg and accuracy of 100 g. If possible, the child was directly weighed. If the baby was too small or cried so hard, first, the mother was weighed alone, and then hugging the child. The scale was automatically calculated the weight of the child by subtracting. Also, every day before starting work, to ensure the accuracy of the scale, the scale was tested using the control scale. The height measuring board was also used to measure height. The height of less than two years children was measured in a supine and larger children were measured in standing position with an accuracy of one tenth in millimeter. The height measuring board was used for both positions. The middle of the left arm circumference in children from 6 to 59 months was measured using a special band of measuring arm circumference based on the following steps and were recorded in millimeters. Chi-square test was used to examine the relationship between variables and malnutrition.

## 3. Results

This study was done on 2525 children under 5 years that, in West Azarbijan, 49.6% of children were boys and 50.4% were girls and the maximum number of children was at 18-29 months (24.7%) and the minimum number was at 54-59 months (8.8%). In Kermanshah, 49% of children were boys and 51% were girls and also, in Isfahan, 50.4% of children were boys and 49.6 were girls (Table 1).

**Table 1:** Distribution of the baseline characteristics of the study population (N=2525)

		Boys		Girls		Total	

		N	%	N	%	N	%
West Azerbaijan	6-17 months	78	45.6	93	54.4	171	19.0
	18-29 months	109	48.9	114	51.1	223	24.7
	30-41 months	114	53.3	100	46.7	214	23.7
	42-53 months	105	48.8	110	51.2	215	23.8
	54-59 months	41	51.9	38	48.1	79	8.8
	Total	447	49.6	455	50.4	902	100.0

Kermanshah	6-17 months	87	47.3	97	52.7	184	22.2
	18-29 months	94	49.2	97	50.8	191	23.0
	30-41 months	93	48.9	97	51.1	190	22.9
	42-53 months	89	49.4	91	50.6	180	21.7
	54-59 months	43	51.2	41	48.8	84	10.1
	Total	406	49.0	423	51.0	829	100.0

Isfahan	6-17 months	93	53.4	81	46.6	174	21.9
	18-29 months	94	48.5	100	51.5	194	24.4
	30-41 months	82	46.1	96	53.9	178	22.4
	42-53 months	93	55.4	75	44.6	168	21.2
	54-59 months	38	47.5	42	52.5	80	10.1
	Total	400	50.4	394	49.6	794	100.0

Generally, malnutrition (Z<-2SD) based underweight was observed in 2.9%, 4.5% and 6.8% of girls in west Azerbaijan, Kermanshah and Isfahan, respectively. Also, 1.6% of boys in west Azerbaijan, 2.8% of girls in Kermanshah and 5.5% of boys in Isfahan were suffer from wasting. In West Azerbaijan 7.7% of girls, 3.8% of girls in Kermanshah and 10.9% of boys in Isfahan were were stunted. The prevalence of underweight and stunting were more common in girls but it was not significant in both sexes (Table 2).

**Table 2:** Prevalence of underweight, stunting and wasting in regions and gender

Area	Gender	Weight for age			Height for age			Weight for height		

		Under weight (≤-2 z-score) (%)	Medium Under weight (<-2, >=-3 z-score) (%)	Severe Under weight (<-3 z-score) (%)	Stunting (<-2z-score) (%)	Medium Stunting (<-2, >=-3 z-score) (%)	Severe Stunting (<-3 z-score) (%)	Wasting (<-2z-score) (%)	Medium wasting (<-2,>=-3 z-score) (%)	Severe wasting (<-3 z-score) (%)
West Azerbaijan	girl	13(2.9)	13(2.9)	0(0)	35(7.7)	29(6.4)	6(1.3)	6(1.3)	5(1.1)	1(0.2)
	boy	8(1.8)	6(1.3)	2(0.4)	31(6.9)	24(5.4)	7(1.6)	7(1.6)	6(1.3)	1(0.2)
Kermanshah	girl	18(4.5)	17(4)	2(0.5)	16(3.8)	14(3.3)	2(0.5)	12(2.8)	11(2.6)	1(0.2)
	boy	12(3)	9(2.2)	3(0.7)	12(3)	11(2.7)	1(0.2)	10(2.5)	8(2)	2(0.5)
Isfahan	girl	28(6.8)	26(6.3)	2(0.5)	43(10.5)	38(9.2)	5(1.2)	17(4.1)	16(3.9)	1(0.2)
	boy	19(4.5)	14(3.3)	5(1.2)	46(10.9)	40(9.5)	6(1.4)	23(5.5)	20(4.8)	3(0.7)

The relationship between gender and region with malnutrition in three provinces showed that there was no statistical difference between sex and underweight, wasting and stunting, but in West Azarbijan, stunting was more common in rural areas and this different was significant (p<0.03) (Table 3).

**Table 3:** Comparing of the prevalence of underweight, stunting and wasting with gender and region in West Azerbaijan

Variable			Weight for age		Height for age		Weight for height	

			Normal (%)	Malnourished* (%)	Normal (%)	Malnourished (%)	Normal (%)	Malnourished (%)
West Azarbijan	gender	girl	443 (95.2)	22(4.8)	411(90.3)	44(9.7)	433(95.2)	22(4.8)
		boy	423(94.6)	24(5.4)	407(91.1)	40(8.9)	416(93.1)	31(6.9)
		P-value		0.72	0.71		0.18	
	Area	urban	405(93.5)	28(6.5)	402(92.8)	31(7.2)	401(92.6)	32(7.4)
		rural	451(96.2)	18(3.8)	416(88.7)	53(11.3)	448(95.5)	21(4.5)
		P-value	0.07		0.03		0.06	
Kermanshah	gender	girl	400(94.6)	23(5.4)	397(93.9)	26(6.1)	404(95.5)	19(4.5)
		boy	386(95.3)	19(4.7)	386(95.1)	20(4.9)	385(95.1)	20(4.9)
		P-value	0.63		0.44		0.76	
	Area	urban	443(94.7)	25(5.3)	443(94.5)	26(5.5)	442(94.6)	26(5.4)
		rural	343(95.3)	17(4.7)	340(94.4)	20(5.6)	347(96.4)	13(3.6)
		P-value	0.69		0.1		0.19	
Isfahan	gender	girl	366(92.9)	28(7.1)	350(88.8)	44(11.2)	373(94.7)	21(5.3)
		boy	381(95.2)	19(4.8)	351(87.8)	49(12.2)	372(93)	28(7)
		P-value	0.16		0.63		0.33	
	Area	urban	455(93.4)	32(6.6)	428(87.9)	59(12.1)	460(94.5)	27(5.5)
		rural	292(95.1)	15(4.9)	273(88.9)	34(11.1)	285(92.8)	22(7.2)
		P-value	0.33		0.66		0.35	

## 4. Discussion

The aim of current study was to determining and comparing the prevalence of malnutrition in children aged less than 5 years in West Azerbaijan, Kermanshah and Isfahan at the same time using WHO, ENA software. Overall, the prevalence of malnutrition based on underweight, stunting and wasting was estimated 2.3%, 7.3% and 1.4% among children in West Azerbaijan, respectively. The results showed that the highest rate of underweight was among Isfahan`s children and the lowest rate was in West Azerbaijan’s children. Also, the rate of stunting and wasting in Isfahan was more than the two other regions while the lowest rate of wasting was observed in West Azerbaijan. Different studies found that malnutrition in children aged less than five years old is varies in Iran. In a study in West Azerbaijan by Eslamlou et al prevalence of underweight, stunting and wasting was estimated 4.3%, 8.7% and 7.5%, respectively (Farrokh-Eslamlou et al., 2013). In another study, the prevalence of malnutrition based on underweight, stunting, and wasting was 7.5%, 12.5% and 4.4%, respectively (Payandeh et al., 2013). According to UNICEF report, 11%, 15%, and 5% of Iranian under five years children suffer from underweight, stunting and wasting up respectively (Veghari, 2012).

It was found that in all three provinces the prevalence of underweight in girls was higher than in boys. Also, according to this study, the prevalence of stunting in girls was more common in West Azerbaijan and Kermanshah. However, in comparison with girls, boys have allocated greater percentage of stunting in Isfahan. In West Azerbaijan and Isfahan, The prevalence of wasting in boys was more than in girls. Comparing the results of this study with the results obtained from national survey of the indices of anthropometric children under 6 years old (2007), it was observed that the results of these studies have been consistent with this report.

The relationship between gender and region with malnutrition in three provinces showed that there was no statistical difference between sex and under-weight, wasting and stunting, but in West Azerbaijan stunting was more common in rural areas and this different was significant.(p<0.03). In current study, prevalence of overweight in West Azarbijan, Kermanshah and Isfahan was 5.1%, 4.5% and 3.7%, respectively. Also, Prevalence of obesity in West Azarbijan, Kermanshah and Isfahan was 1.3%, 0.7% and 0.1%, respectively. Prevalence of obesity and overweight in boys and urban areas was more than girls and rural areas but this difference was not significant. Prevalence of overweight and obesity among boys in urban areas were more common in West Azerbaijan. In this area stunting, overweight and obesity are the most important priorities that health officials should pay more attention to this problem.

Given the differences between various provinces and regions of the country which are as a result of the differences between the levels of development in these areas, the necessity of designing and implementing targeted strategies are required for different areas. It is worth noting that the present study has been conducted in a single period and in only one city of each province and using ENA software; therefore, the judgment about the whole province requires general investigation in all cites of provinces and the obtained results are solely applied to these three cities. This study, also, showed that the ENA software has a special ability to determine the samples and clusters and is a simple, rapid and accurate method, especially in epidemiological studies in the country can be a convenient tool and its use is suggested for the same studies. Also, the quality control of the performed activities by the teams in the field is another distinctive feature of this software which is considered of high importance and emphasizes the use of this software.
